# The anterior insula and anterior cingulate cortex are associated with avoidance of dental treatment based on prior experience of treatment in healthy adults

**DOI:** 10.1186/s12868-015-0224-9

**Published:** 2015-12-10

**Authors:** Chia-Shu Lin, Shih-Yun Wu, Long-Ting Wu

**Affiliations:** Department of Dentistry, School of Dentistry, National Yang-Ming University, No. 155, Sec. 2, Linong Street, Taipei, 11221 Taiwan, ROC; Division of Family Dentistry, Department of Stomatology, Taipei Veterans General Hospital, Taipei, Taiwan, ROC; Division of Endodontics and Periodontology, Department of Stomatology, Taipei Veterans General Hospital, Taipei, Taiwan, ROC

**Keywords:** fMRI, Anterior insula, Anterior cingulate cortex, Dental fear, Dental avoidance

## Abstract

**Background:**

Fear concerning stressful medical or dental procedures is one of the major factors that distance patients from health care. Fear and avoidance of dental treatments can be shaped by a patient’s prior experience with receiving dental procedures or by imagining the procedures.

**Methods:**

We performed two functional magnetic resonance imaging (fMRI) experiments to investigate the role of the anterior insula (aINS) and dorsal anterior cingulate cortex (dACC), which are both critical to threat perception, in dental avoidance. Dental avoidance based on both prior treatment experience and imagination was assessed using a customized questionnaire. In an fMRI task-based study, we investigated brain activation in 17 healthy participants when they viewed images depicting dental procedures that evoked a moderate degree of fear. Region-of-interest analysis was performed to assess the association between dental avoidance and aINS as well as dACC activation. In a resting state fMRI study, we investigated 18 healthy participants for the association between the intrinsic functional connectivity of the aINS and dACC and dental avoidance.

**Results:**

We found that (1) the participants showed a higher activation of the right aINS and bilateral dACC when they viewed images of dental procedures compared with the brain activation observed when they viewed scrambled images (*p* < 0.05 corrected for small volume and family-wise error). (2) The avoidance ratings based on prior experience of dental treatment were significantly positively correlated with the activation in the right aINS (*r* = 0.67, *p* = 0.003), right dACC (*r* = 0.65, *p* = 0.005) and left dACC (*r* = 0.63, *p* = 0.007). (3) The intrinsic functional connectivity between the aINS and the orbitofrontal cortex was positively correlated with the avoidance ratings based on experience (uncorrected *p* < 0.001).

**Conclusions:**

The findings highlight prior experience of dental treatment as a predominant factor in shaping patients’ avoidance behavior. Individual differences in threat perception may play a key role in the development of dental avoidance.

## Background

Fear concerning stressful medical or dental procedures, such as gastrointestinal endoscopy, bronchoscopy or wisdom tooth extraction, is one of the major factors that distances patients from health care [1–4]. The fear of dental treatment is strongly associated with avoidance of dental treatment [5, 6], which results in deteriorated oral health, potentiates the further fear of treatment, and reduces a patient’s willingness to receive treatment [2, 6]. Fear and avoidance can be derived from one’s experience of prior dental treatment, such as a prior traumatic treatment experience being associated with increased fear toward treatment [7, 8], or it can be due to an imagined fear that is typically shaped by verbal instruction (e.g., messages from the media) or observation [9]. However, it remains unclear how the experience of prior dental treatment influences avoidance because the underlying neural mechanisms have not been fully investigated.

Cumulating evidence has revealed that anterior insula (aINS) and dorsal anterior cingulate cortex (dACC) activation is associated with fear and avoidance. First, research on dental phobia has revealed that visual (e.g., images depicting dental procedures), auditory (e.g., drilling sounds) or cross-modal materials (e.g., video of dentistry actions) [10–14] evoke fearful experiences and heightened activation at both the dACC and aINS [10, 13], together with the orbitofrontal cortex (OFC) [10, 12, 13]. Second, aINS and dACC activation has been associated with painful experiences when the subjects imagined [15–17] or recalled a painful experience [18–20], even without actually receiving the stimuli. Third, research on fear-defensive behavior has revealed that increased dental fear is associated with heightened defensive reactivity in the presentation of a painful threat [21]. Both the dACC and aINS are critical components in the defensive fear system [22], and activation was found in both the aINS and dACC when subjects anticipated or encountered a threatening object [23, 24]. The convergent findings suggest that the aINS and dACC may play a key role in dental fear and avoidance. Finally, long-term experience is able to shape brain function and structure, an effect known as neuroplasticity [25], and altered brain connectivity of the aINS and the dACC is associated with long-term psychological stress [26, 27]. Therefore, aINS and dACC connectivity may be associated with the degree of dental avoidance, which could be associated with a prior stressful experience [7, 28, 29].

The current study aimed to investigate the neural correlates of dental avoidance by using functional magnetic resonance imaging (fMRI). Two fMRI studies were performed to test the following hypotheses regarding the neural correlates underlying dental avoidance:

1. In the task-based fMRI study, we used visual stimuli to evoke fear about dental treatment. We hypothesized that brain activation of the aINS and the dACC would be positively correlated with the degree of dental avoidance.
2. In the resting-state fMRI study, we investigated whether there was an association between the intrinsic functional connectivity and the individual differences in dental avoidance. We hypothesized that aINS and dACC connectivity would be positively correlated with the degree of dental avoidance.

## Methods

### Participants

Twenty-one healthy participants were recruited from a university campus via bulletin board notices. All participants were screened for the following exclusion criteria: (1) having a history of major physical or psychiatric disorders including epilepsy, major depression, schizophrenia or neurovascular diseases, (2) having a history of brain injury or having undergone brain surgery, and (3) being unable to undergo MRI due to physical (e.g., having a surgical implant) or psychological (e.g., claustrophobia) contraindications. Seventeen participants (eight females, age 24.2 ± 2.0 (mean ± standard deviation)) participated in the ‘dental threat’ fMRI experiment. Eighteen participants (nine females, age 24.5 ± 2.1) participated in the ‘resting-state’ fMRI experiment. Fourteen of the 21 participants participated in both experiments. None of the participants had a history of major physical or psychiatric disorders or a history of chronic orofacial pain as assessed by a dentist (the first author, C-S L). Notably, because the current study aimed to investigate the neural correlates associated with the degree of dental avoidance, we did not exclusively recruit the participants with extreme dental fear, as done in previous studies [10, 12]. Therefore, the participants here represented a group of both low dental anxiety and high dental anxiety patients (see Results). All participants provided written informed consent before participating in this study. The study conforms to the ethical standards presented in the Declaration of Helsinki, and the study protocol was approved by the Institutional Review Board of Taipei Veterans General Hospital (VGHIRB No. 2013-06-026BY).

The number of participants was determined based a power analysis on the behavioral findings. Because we focused on the strength of association between the regional brain activation and the behavioral score (i.e., dental avoidance), we performed a power analysis to evaluate the sample size for a one-tailed bivariate correlation analysis with alpha = 0.05, beta = 0.8, and effect size (r) = 0.6. The effect size was estimated according to the previous findings [12], which showed the fear experience evoked by video was positively correlated with activation of the insula, the thalamus and the anterior cingulate, with r between 0.58 and 0.69. The analysis was performed using G*Power v3.1.9.2 [30] and showed a minimum of N = 15.

### Experimental paradigm

The study consisted of two fMRI experiments. Seventeen participants completed a task-based ‘dental threat’ experiment. Dental avoidance based on an experience of prior treatment (i.e., experience-based avoidance) and imagination (i.e., imagination-based avoidance) was assessed using a customized questionnaire (Table 1). Eighteen participants completed a resting-state experiment.

**Table 1:** Customized questionnaire for dental avoidance

No.	Dental procedure^a^	(A) Having received the procedure? (yes/no)	(B) Avoidance: 0–10
1	Receiving a local anesthetic injection in the mouth		
2	Having a painful tooth tapped by the dentist		
3	Having a primary tooth (milk teeth) extracted in the childhood		
4	Receiving ultrasonic scaling for removing dental stone		
5	A molar being drilled to remove caries		
6	Receiving a root canal treatment		
7	Having a wisdom tooth extracted by surgery		
8	Feeling painful hypersensitivity when rinsing cold water		
9	A caries tooth being explored with a dental instrument		
10	Having the swelling gum incised and pus drained		
11	Feeling excruciating postoperative pain; not being relieved even with painkillers		
12	Receiving a wedge and band in between the teeth during restoration		

Instruction: For each of the following dental procedures, please indicate if you have received it or not, by marking ‘Yes’ or ‘No’ in the column (A), and rate the degree that you would avoid receiving the procedure in the column (B). The rating should be given by a number between 0 and 10 (0 = the least/10 = the maximal degree). If you have received the procedure before, please rate avoidance based on your past experience of the procedure. If you have not experienced the procedure, please rate avoidance based on your imagination about it

^a^The items No. 1, 5, 6, 7, 8, 9, 10, 11 were modified from the Fear of Dental Pain questionnaire

^b^Scoring method: *Experience*-*based avoidance* = mean avoidance ratings (B) from the items that are marked as ‘Yes’ in (A); *Imagination*-*based avoidance* = mean avoidance ratings (B) from the items that are marked as ‘No’ in (A)

### The ‘dental threat’ experiment

In the ‘dental threat’ study, we used visual images to elicit fear toward dental treatment. Such a paradigm has been adopted in previous studies concerning dental phobia [11, 12]. During the fMRI scan, 17 participants viewed a set of dental procedure images and performed the following tasks:

- In the ‘Re-experiencing’ task (REXP), the participants were instructed to imagine that they were receiving the dental procedure and to imagine the pain or fear that they would experience from it. They were instructed to ‘*Imagine that you are the subject who is receiving this treatment, and imagine the pain or fear that you would perceive, related to this treatment.*’ The participants were asked to take a self-perspective as if they were in the same situation as depicted by the image [18] and to immerse themselves with these unpleasant feelings.
- In the ‘counting’ task (COUNT), the participants were instructed to count how many teeth they could find in the image. They were instructed to ‘*Count how many teeth that you find in this image.*’
- In the ‘scrambled image’ task (SCM), the participants were instructed to view a set of images displayed in the counting task that were scrambled as a visual control.

**Fig. 1:**
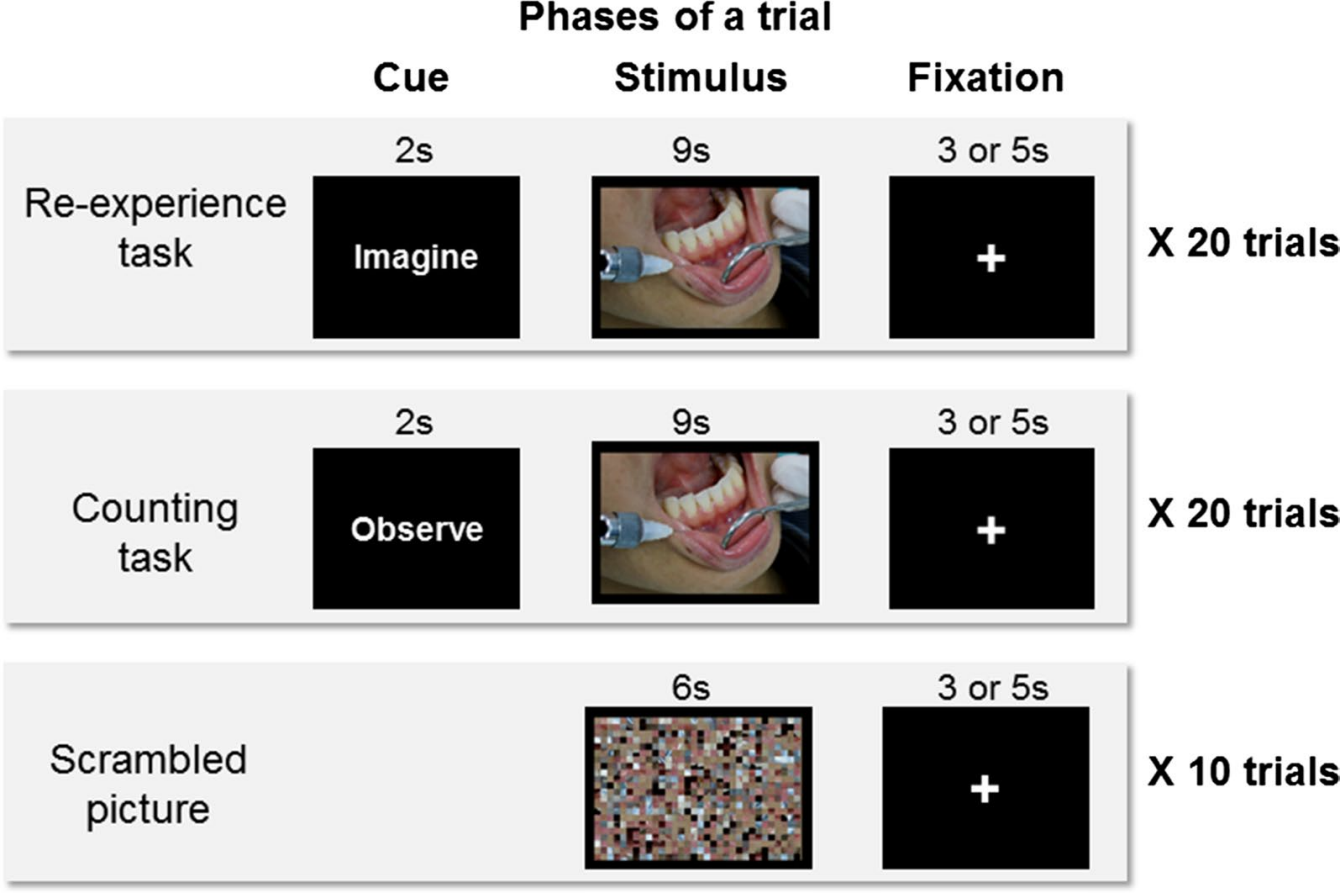
Experimental paradigm. During the fMRI scan, the participants were asked to perform re-experience tasks (REXP, 20 trials), counting tasks (COUNT, 20 trials) and visual control tasks (SCM, 10 trials). The REXP and the COUNT tasks consisted of three phase: (1) cue (2 s), (2) stimulus (9 s for the REXP and the COUNT tasks and 6 s for the SCM task), and (3) fixation (3 or 5 s)

The whole experiment consisted of 20 REXP trials, 20 COUNT trials and 10 SCM trials, for a total of 50 trials (Fig. 1). Each REXP or COUNT trial consisted of three phases. In the initial *cue* phase, the word ‘imagine’ or ‘observe’ was displayed for 2 s to instruct the participants to perform the REXP or COUNT task, respectively, in the subsequent phase. In the *stimulus* phase, the image depicting the dental procedures was displayed for 9 s, and the participants performed a REXP or COUNT task according to the instruction given in the preceding cue phase. Finally, in the *fixation* phase, the participants were instructed to look at a fixed cross that was displayed for 3 or 5 s.

### The ‘resting-state’ experiment

During the fMRI scan, 18 participants were instructed to be relaxed and remain awake with eyes open. They were instructed to fix their eyes on a cross symbol on the screen.

### Preparation of images

Twenty images depicting 7 categories of common dental procedures including tooth extraction (*N* = 4), local anesthesia injection (*N* = 3), having a tooth surface polished (*N* = 4), drilling a tooth (*N* = 2), probing the gum (one image for the anterior site and one for the posterior site) (*N* = 2), exploring an anterior tooth (*N* = 4) and scaling an anterior tooth (*N* = 1) were created by photographing a healthy female subject who was not a study participant. All of the images were matched for brightness and hue using Adobe Photoshop^®^ 7.0 (Adobe Systems, San Jose, California). The scrambled images were generated with a scramble filter tool (http://www.telegraphics.com.au/sw/). It should be noted that in this study, we aimed to elicit increased perceived threat by asking the participants to recall their past experiences of dental treatment rather than to directly evoke a strong aversive experience, such as disgust. Therefore, we photographed a subject with relatively good oral hygiene to minimize the potential feeling of disgust.

### Assessment of behavioral data

#### Dental avoidance and trait dental anxiety

Dental avoidance was assessed using a customized self-report questionnaire that consisted of 12 dental procedure scenarios (Table 1). The dental procedures and descriptions were selected based on the fear of dental pain questionnaire (FDP) [31]. The participants were asked to indicate the degree of avoidance to each item using the previously described method [8]. For each participant, we distinguished the avoidance based on his/her past treatment experience from the avoidance based on imagination. For each scenario, the participants were required to (1) mark if they had received the procedure or not and (2) rate the degree to which they would avoid receiving the procedure. For each scenario, the participants were instructed to rate their avoidance based on their past experience about the procedure if they had received the procedure previously, or to rate avoidance based on their imagination if they had not experienced that procedure. The mean avoidance rating from the experienced procedures was indexed as the *experience*-*based avoidance*, and the mean avoidance rating from the non-experienced procedures was indexed as the *imagination*-*based avoidance*. All ratings were based on an 11-point numerical rating scale (NRS, 0 = the least degree and 10 = the maximal degree). Trait dental anxiety was assessed using the modified dental anxiety scale (MDAS) [32]. The Chinese version of the MDAS was used in the current study [33]. Its psychometric properties have been reported in a previous study, based on a Chinese-speaking sample [33]. The MDAS assessment was performed before the MRI scan. Statistical analysis was performed using Minitab version 16.1.0 (Minitab, Inc., State College, Pennsylvania).

#### Fear elicited by dental procedures

After the fMRI scan, the participants immediately rated how threatened they felt with respect to the images that they observed during the fMRI scan based on an 11-point numerical rating scale (NRS, 0 = not threatening at all and 10 = extremely threatening). The rating was indexed as the *elicited fear*. The participants were asked to briefly describe the dental procedures depicted by each image.

### Acquisition and pre-processing of imaging data

#### The ‘dental threat’ experiment

The data were acquired on a 3-Tesla imaging system (Tim Trio, Siemens, Erlangen, Germany) with a quadrature head coil. Functional data were acquired with T2-weighted gradient-echo EPI using blood-oxygenation-level-dependent contrast (TR/TE/flip angle = 2000 ms/20 ms/90°) with the following parameters: matrix size = 64 × 64 × 40 and a voxel size = 3.4 × 3.4 × 3.4 mm^3^. The total scan time was 6.5 min. An anatomical image was acquired using a T1-weighted 3D gradient-echo pulse sequence (modified driven equilibrium Fourier transform: TR/TE/TI = 2530/3.03/1100 ms) with the following parameters: matrix size = 256 × 256 × 192 and a voxel size = 1×1 × 1 mm^3^.

Functional imaging data were pre-processed and analyzed using Statistical Parametric Mapping (SPM8, the Wellcome Trust Centre for Neuroimaging, London, http://www.fil.ion.ucl.ac.uk/spm). The following protocol for pre-processing was based on our published study [34]. Scans were slice-time corrected, realigned and co-registered to the individual T1-weighted anatomical image before being normalized to a 2 × 2 × 2 mm MNI152 (Montreal Neurological Institute) space. Scans were further smoothed using a FWHM (full width at half maximum) 8 × 8 × 8 mm Gaussian kernel, high-pass filtered, and corrected for temporal serial correlations.

#### The ‘resting-state’ experiment

The data were acquired on the same scanner with the following parameters: [TR]/[TE]/flip angle = 2500 ms/30 ms/90°; matrix size = 64 × 64 × 40; voxel size = 3.4 × 3.4 × 3.4 mm^3^. The total scan time was 8.3 min. Pre-processing was performed using the Data Processing Assistant for Resting-State fMRI (DPARSF, http://www.restfmri.net) and the Resting-State fMRI Data Analysis Toolkit (REST, http://www.restfmri.net) based on the SPM8 software. The following pre-processing protocol was based on our published study [35]. All scans were slice-time corrected, corrected for head movement and normalized to the Montreal Neurological Institute (MNI) template. The time series from the seed region was band-pass filtered (0.01–0.1 Hz) to extract the low-frequency oscillating components that contributed to resting-state functional connectivity. The six movement parameters of rigid body translation and rotation and the mean signal of the cerebrospinal ventricles and the deep white matter were removed as nuisance regressors using multiple regression. Global signal regression was performed to reduce the influence of noise introduced by physiological activities.

### Analysis of imaging data

#### The ‘dental threat’ experiment

At the individual level, we modeled the cue phase for 2 s, the fixation phase for 3 or 5 s, and the stimulus phases for 6 s (starting from the 4th second to the end of the phase) using a canonical hemodynamic response function. Head movement parameters were modeled as the regressors of no interest. A random-effect analysis was performed at the group level: a one-sample *t* test was performed for the first-level contrasts REXP > SCM and COUNT > SCM across all participants. An exploratory whole-brain analysis was performed by thresholding with an uncorrected *p* < 0.001 and a cluster size >10 voxels. Based on our primary hypothesis, we performed a region of interest (ROI)-based analysis with small volume correction (SVC) on the bilateral aINS and dACC ROIs. The masks of these ROIs were defined according to a previous study [36]. The results were considered statistically significant at *p* < 0.05 and were corrected for family-wise error (FWE). To quantify the strength of association between dental avoidance the aINS as well as dACC activation, we calculated the Pearson correlation coefficient between the regional activation of the aINS and the dACC ROIs and the ratings of experience- and imagination-based avoidance across all participants. Regional activation was quantified as the mean beta value from all of the voxels within the ROIs of the contrast REXP > SCM. The Pearson correlation coefficient was considered statistically significant at *p* < 0.05 (two-tailed test of significance).

#### The ‘resting-state’ experiment

We performed a seed-based functional connectivity (SBFC) analysis based on published methods [37]. The analysis revealed the functional connectivity related to the seed and was quantified as the correlation of brain activity patterns between the seed region and other parts of the brain. The aINS and dACC were used as the seed regions. The masks of the seed regions were defined based on the results from the dental threat experiment. Regression analysis was performed using the ratings of experience- or imagination-based avoidance as the covariate to reveal the brain region in which connectivity with the seed region was significantly correlated with the experience- or imagination-based avoidance. For an exploratory purpose, all imaging results were thresholded at an uncorrected *p* < 0.001, with a cluster size >10 voxels.

## Results

### Demographic and behavioral data

**Fig. 2:**
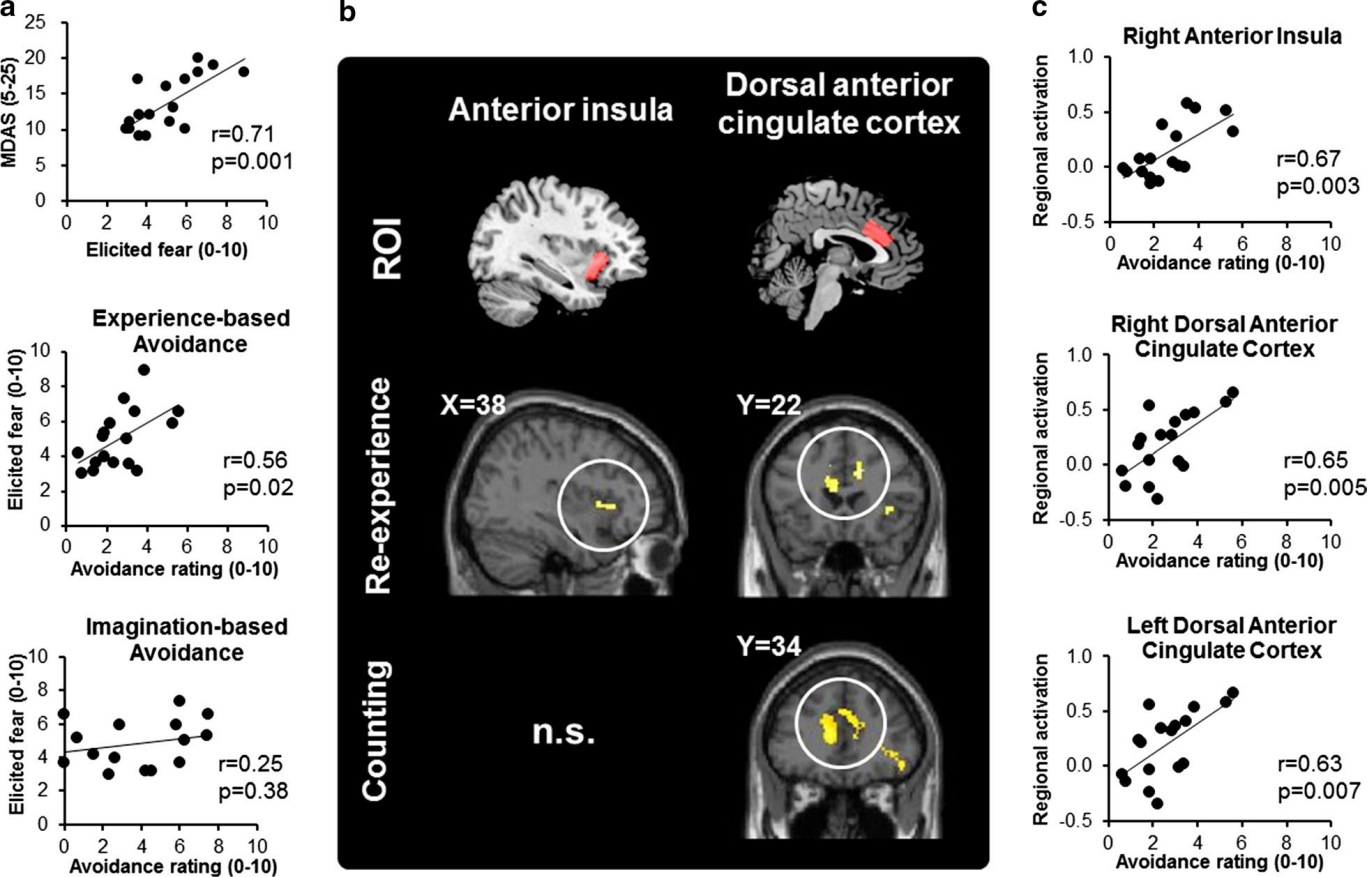
**a** The ratings of elicited fear were significantly correlated with the MDAS score and avoidance ratings based on experience. No significant correlation was found between the ratings of elicited fear and the avoidance ratings based on imagination. **b** Results of the ROI-based analysis. The anterior insula (aINS) and the dorsal anterior cingulate cortex (dACC) were selected as the regions of interest (ROIs) (the *upper panel*). The REXP task showed an increased activation in the right aINS and the bilateral dACC (the *middle panel*). The COUNT task showed an increased activation in the bilateral dACC (the *lower panel*). A cluster was considered statistically significant by small volume-correction based on the pre-defined ROIs (*p* < 0.05 corrected for family-wise error). **c** The ratings of experience-based avoidance were significantly correlated with the activation in the right aINS, the right dACC and the left dACC during the re-experience task

The demographic and behavioral data are summarized in Table 2. The customized self-report questionnaire of dental avoidance was evaluated for internal consistency using Cronbach’s alpha and for test–retest reliability using intraclass consistency (ICC). The assessment showed both good internal consistency (Cronbach’s Alpha = 0.90) and a test–retest reliability of 0.91. The scores of experience-based avoidance, imagination-based avoidance, the modified dental anxiety scale (MDAS), and elicited fear all followed a normal distribution as assessed by the Kolmogorov–Smirnov test for normal distribution. Across all participants, the avoidance ratings based on imagination were slightly greater than ratings based on experience, but the difference was not statistically significant (paired t-test *p* > 0.05 for both experiments) (Table 2A, B). In the ‘dental threat’ experiment, the participants reported a moderate degree of elicited fear (mean ± standard deviation = 5.0 ± 1.7) (Table 2A). A post-experiment survey revealed that all the participants were able to recognize the dental procedures depicted in the images. The ratings of elicited fear were significantly positively correlated with the MDAS score (*r* = 0.71, *p* = 0.001) (Table 2C; Fig. 2a), which indicated that the visual stimuli successfully elicited fear with respect to receiving dental procedures. The MDAS score showed a moderate degree of trait dental anxiety (mean ± standard deviation = 13.6 ± 3.9, min/max = 9/20) among the participants (Table 2A). Seven participants showed low dental anxiety (MDAS score 5–11), 8 showed moderated dental anxiety (MDAS score 12–18) and 2 showed high dental anxiety (MDAS score 19–25) based on the published cut-off points [32, 38]. The findings suggested that both low- and high-anxiety persons were included. Importantly, elicited fear was significantly positively correlated with the ratings of experience-based avoidance (*r* = 0.55, *p* = 0.02) but not with ratings of imagination-based avoidance (*r* = 0.25, *p* = 0.38) (Table 2B; Fig. 2a), which suggests a close association between fear and prior experience of dental treatment.

**Table 2:** Demographic and behavioral data

	Mean	SD	Min	Max	*p* value^a^
(A) ‘Dental fear’ experiment (n = 17)*					
Gender (male/female)	9/8				
Age (years)^b^	24.2	2.0	20	27	
Dental avoidance^2^					
Experience-based	2.6	1.4	0.6	5.6	0.06
Imagination-based^c^	3.8	2.6	0	7.5	
MDAS score^b^	13.6	3.9	9	20	
Elicited fear^b^	5.0	1.7	3.0	8.9	
(B) ‘Resting state’ experiment (n = 18)					
Gender (male/female)	9/9				
Age (years)^b^	24.5	2.1	22	29	
Dental avoidance^2^					
Experience-based	2.5	1.7	0.8	5.9	0.07
Imagination-based	3.4	2.4	0	7.5	

	Dental avoidance		MDAS score	Elicited fear
	Experience-based	Imagination-based		
(C) Correlation of the variables from the ‘Dental fear’ experiment				
Age (years)	−0.298	−0.405	−0.123	0.020
Dental avoidance				
Experience-based		0.396	0.763***	0.551*
Imagination-based			0.344	0.250
MDAS score				0.711**

*MDAS* Modified Dental Anxiety Scale, *SD* standard deviation

* Denotes the p value of two-tailed Pearson correlation coefficient <0.05; ** p value < 0.01; *** p value <0.001

^a^Two-tailed paired t-test was performed from comparing between the ‘experience-based’ and the ‘imagination-based’ scores

^b^Normality was assessed according to the the Kolmogorov–Smirnov test for normal distribution; normality was accepted if *p* > 0.05. The patients’ age, the scores of dental avoidance, MDAS, and elicited fear conformed to normal distribution

^c^Two participants did not complete the dental avoidance ratings based on imagination

### Results of the dental threat experiment

The findings of the ROI-based analysis are shown in Fig. 2b. When the participants viewed the treatment-related images and re-experienced their past experience of treatment (i.e., the re-experiencing task, REXP) compared with viewing the visual control (i.e., the scrambled image task, SCM), we found an increased activation in the right anterior insula (aINS) ([*x,y,z*] = [38,20,0], size = 23 voxels, *Z* = 3.46, *p*_FWE-corrected_ = 0.01), in the right dACC ([*x,y,z*] = [12, 22, 30], size = 7 voxels, *Z* = 3.82, *p*_FWE-corrected_ = 0.004), and in the left dACC ([*x,y,z*] = [−6,22,20], size = 15 voxels, *Z* = 3.38, *p*_FWE-corrected_ = 0.015). When the participants counted the number of teeth (i.e., the counting task, COUNT) compared with the visual control, there was an increased activation in the bilateral dACC (Fig. 2b). In the REXP task, the post hoc correlation analyses revealed that the rating of experience-based avoidance was significantly correlated with brain activation in the right aINS (*r* = 0.67, *p* = 0.003), right dACC (*r* = 0.65, *p* = 0.005) and left dACC (*r* = 0.63, *p* = 0.007) (Fig. 2c). In contrast, in the COUNT task, the correlation was not statistically significant. The findings confirmed our hypothesis that aINS and dACC activation is associated with the degree of dental avoidance. The data from the exploratory whole-brain analysis are shown in Table 3.

**Table 3:** Clusters of significant brain activation in the re-experience and the counting tasks

Brain region	Side	Cluster size (voxel)	*Z* score	MNI coordinates		
				*x*	*y*	*z*
Re-experience (REXP) task						
dACC	L	256	3.6	−10	24	36
dACC	R	57	3.9	12	20	30
dACC	R		3.2	14	24	38
dACC	R	78	3.7	12	38	26
dACC	R		3.3	10	32	34
MTG/STG	L	22	3.5	−52	−32	−2
MTG/STG	L		3.2	−58	−26	−6
aINS	R	33	3.5	38	20	0
aINS	R		3.3	38	12	4
Inferior parietal lobule	L	25	3.4	−50	−70	36
Lingual gyrus	L	14	3.3	−26	−52	4
aINS	L	14	3.3	−34	4	4
Counting (COUNT) task						
dACC	L	454	4.4	−12	32	28
dACC	L		4.0	−8	40	12
dACC	L		3.6	−12	34	20
lOFC	L	57	4.0	−50	32	−8
lOFC	R	170	4.0	44	32	−8
lOFC	R		3.8	38	30	−16
lOFC	R		3.6	36	42	−2
Frontal Pole	R	246	3.9	12	46	32
dACC	R		3.8	12	42	22
dACC	R		3.6	2	38	32
Superior parietal lobule	R	17	3.8	6	−64	70
Frontal pole	R	62	3.8	32	56	−14
Frontal pole	R		3.5	32	48	−16
Lateral occipital cortex	R	49	3.7	32	−68	8
Superior frontal cortex	L	16	3.6	−10	28	50
Superior parietal lobule	L	26	3.6	−4	−56	74
Superior parietal lobule	R		3.4	6	−52	76
Lateral occipital cortex	L	52	3.5	−56	−66	28
Inferior parietal lobule	L		3.5	−64	−54	24
MTG	R	34	3.4	64	−22	−6
Lateral occipital cortex	L	14	3.3	−34	−62	4

All results reported are uncorrected for multiple comparison. Cluster size is measured by the number of voxels

*aINS* anterior insula, *dACC* dorsal anterior cingulate cortex, *lOFC* lateral orbitofrontal cortex, *MTG* middle temporal gyrus, *STG* superior temporal gyrus

It should be noted that the association between experience-based dental avoidance and brain activation, as reported above, can be confounded by the patient’s age and MDAS score (Table 2A). We therefore performed multiple regression analyses by modeling the regional activation as the dependent variable and the experience-based dental avoidance, age and the MDAS score as the predictors. The analyses showed that the avoidance ratings were the only significant predictor of brain activation in the ROIs (right aINS: *p* = 0.017; right dACC: *p* = 0.029; left dACC: *p* = 0.046).

### Results of the resting-state experiment

**Fig. 3:**
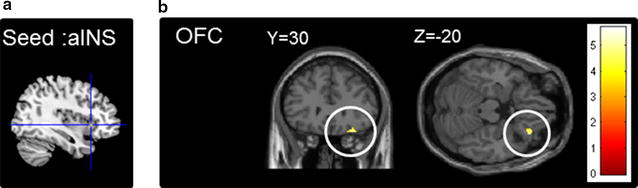
**a** The right anterior insula (aINS) was used as the seed region for the seed-based functional connectivity analysis. The region was defined as a sphere (diameter = 6 mm) centered at [*x, y, z*] = [38, 20, 0]. **b** The whole-brain exploratory analysis revealed that the connectivity between the right aINS and the right orbitofrontal cortex was significantly correlated with the ratings of recalled avoidance

When the right aINS was used as a seed (Fig. 3a), we found that its connectivity with the right orbitofrontal cortex (OFC) was positively correlated with the ratings of experience-based avoidance ([*x, y, z*] = [28, 30, −20], size = 33 voxels, *Z* = 3.77, *p*_uncorrected_ < 0.001) (Fig. 3b). No significant correlation was found between the right aINS-OFC connectivity and the ratings of imagination-based avoidance. The findings suggested that the connectional changes of the threat-related region (i.e., aINS) reflected the degree of dental avoidance that was shaped by prior experience of dental treatment. No significant finding was found when the dACC was used as a seed.

## Discussion

### Summary of the major findings

Dental avoidance is closely associated with the fear of dental treatment. It can be shaped by a patient’s past experience with respect to receiving dental procedures or by imagination that is primarily derived from verbal or observational learning. Using two fMRI studies, we have demonstrated the following neural mechanisms underlying dental avoidance:

1. When fear was evoked by viewing images of dental procedures, the participants showed greater activation of the right aINS and bilateral dACC (Fig. 2b).
2. The aINS and dACC activation was positively correlated with dental avoidance based on experience but not dental avoidance based on imagination (Fig. 2c).
3. In the resting state, the intrinsic functional connectivity between the aINS and OFC was positively correlated with dental avoidance based on experience but not with dental avoidance based on imagination (Fig. 3c).

In sum, these findings revealed that the activation of the aINS and dACC, which are both critical to threat perception, is associated with the ratings of dental avoidance.

### Dental avoidance and activation of the aINS and the dACC

In the ‘dental threat’ study, we found an increased activation in the aINS and dACC during the re-experience task, which evoked a moderate degree of fear (Fig. 2b). The significant correlation between the experience-based dental avoidance and the aINS as well as dACC activation (Fig. 2c) may reflect a heightened perceived threat when the participants viewed the treatment-related images. Both the aINS and dACC are critical to integrating salience signals (e.g., a painful procedure) from a threatening context, and the activation of these regions is associated with the individual difference in pain perception [39]. The aINS activation reflects changes in not only emotional experience but also interoception, i.e., awareness of one’s internal bodily states [40]. Over-prediction of an aversive bodily state may potentiate anxiety and avoidance behavior [41]. Therefore, the aINS activation reported here may reflect the emotional awareness of a negative experience (e.g., fear) and an increased interoceptive experience (i.e., pain) related to treatment. The dACC may play an additional role in both avoidance behavior and the decision-making process [22, 42, 43]. When facing a threatening object, the dACC is associated with the selection of an action in response to the stimuli. In contrast, the aINS is associated with the awareness of threat [42]. Therefore, our findings suggest that the individual difference in threat perception may play a key role in the development of dental avoidance.

Our findings echoed the previous reports about the neural mechanisms of a specific phobia. For example, dental phobia was associated with heightened activation in the dACC and aINS when the subjects were viewing images or videos depicting dental procedures [10, 13]. In patients with spider phobia, dACC activation was associated with the degree of the visual avoidance of the images of spiders [44]. Our findings further suggest that pain is the primary negative experience that dental patients would choose to avoid. The activation of both aINS and dACC is associated with the painful experience elicited by viewing images depicting injury and replaying one’s past experience of pain [15, 18]. Increased dACC activation was found when subjects escaped from painful stimuli, which was an imminent threat [23]. Notably, right aINS activation was found exclusively during the REXP task. The results echoed a recent finding that aINS activation was found when the participants recalled their pain experience [19].

### Functional connectivity related to dental avoidance

Neuroimaging evidence has shown that changes in aINS-dACC connectivity are associated with post-traumatic stress disorder (PTSD) and panic disorder [26, 27]. These disorders are characterized by a heightened perceived threat in the patients. The intrinsic functional connectivity between the anterior cingulate cortex and the aINS was positively correlated with the score of harm avoidance, which is a marker of trait anxiety [45]. In our study, however, we did not find a change in aINS-dACC connectivity, as shown in the previous studies. A possible explanation is that psychological stress is milder in the case of dental visits than is the stress related to PTSD or panic disorders. Therefore, the effect of plasticity is less significant than it is in patients with mental disorders.

Still, we found that aINS-OFC connectivity was associated with the individual difference in dental avoidance. Structurally, the aINS is predominantly connected to the OFC [46], and the projection from the insula to the OFC may convey somatosensory information [47]. Functionally, the OFC is a critical region related to anxiety personality [48], particularly for fear related to pain [49]. The OFC also subserves the emotion-laden process of decision-making [50]. The insula, as the core of the intrinsic salience network, may integrate the signal for a high-salience object from a threatening context (e.g., viewing images depicting painful procedures) [51]. Therefore, aINS-OFC connectivity may represent the integration between threat perception and decision-making with respect to dental visits.

### Limitations of the study

The findings should be cautiously interpreted due to some limitations of the experimental design. First, we did not perform an in-scan trial-by-trial rating of the treatment-related experience for each experiment trial. We considered that the in-scan rating procedure may interfere with the re-experiencing task and that the additional rating procedure may prolong the total scanning time. The trade-off is that we were not able to trace dynamic changes in the emotional status. Second, we did not assess the degree of vividness or accuracy of the treatment-related experience. Memories of traumatic events, such as receiving a stressful dental procedure, may be vulnerable to false memories [52]. For example, the participants may misattribute a painful experience, which was actually elicited by a root canal treatment, to a third molar extraction. In the current study, we did not separately investigate each of the 12 dental procedures. Instead, we pooled the ratings from the 12 dental procedures and formed an overall score on fear and avoidance.

## Conclusions

The results from our experiments revealed that the regional activation and connectional changes related to the aINS and dACC were associated with the individual differences in dental avoidance. The findings highlighted that prior dental experience as a predominant factor in shaping patients’ avoidance behavior and that the individual differences in threat perception may play a key role in the development of dental avoidance.
